# Joint Adaptation of a Digital Mental Health Intervention for University Students: Inductive Qualitative Analysis

**DOI:** 10.2196/80776

**Published:** 2026-01-06

**Authors:** Jemima Dooley, Nouf Alsaadi, Edward Watkins

**Affiliations:** 1 Department of Psychology, Sir Henry Wellcome Centre for Mood Disorders Research, University of Exeter, Perry Road, Exeter, Devon, EX4 4QQ, United Kingdom, 441392726449, ext 6647; 2 School of Education, St Luke’s Campus, University of Exeter, Exeter, United Kingdom

**Keywords:** youth mental health, co-design, co-development, university students, digital platforms

## Abstract

**Background:**

Digital mental health interventions (DMHIs) can be particularly effective for young people, who live more of their lives online than older generations. Co-designing mental health support with young people can combat the challenges of a lack of engagement and sustained use. While this is increasingly common, there are often budget and timeline restraints in research settings that limit true co-design. As part of the Nurture-U project exploring a whole-university approach to student mental health, we coadapted an existing digital platform, i-Spero (P1Vital), with university students. This paper is a reflection on the impact that our student advisors had on the end product, and where the guidance of the young people was implemented, and not implemented, within the existing research parameters.

**Objective:**

This study aims to present an inductive analysis of meeting notes and recordings of the co-design process, in order to highlight what aspects of DMHIs our advisors valued and what, as a research team, we were able to implement. The hope is that this will inform future mental health interventions in this age group.

**Methods:**

The i-Spero digital well-being platform was developed over an iterative process with multiple rounds of feedback from student advisors in 2022-2024. An inductive qualitative analysis approach was implemented by 2 authors (NA and JD) on the detailed feedback reports and meeting summaries of this process to generate categories and themes from the student advisors’ feedback.

**Results:**

Three themes were created: “Relevance and Usefulness,” highlighting the importance of comprehensive features linking in with all aspects university life, while treating young people as adults; “Simplicity and Clarity,” with student advisors suggesting edits that removed burden from the user and eased access to support; and “Acceptability and Inclusiveness,” ensuring awareness of the needs of students from different backgrounds, and what young people with mental health difficulties may be able to access in times of need.

**Conclusions:**

There are some challenges in ensuring that DMHIs are both comprehensive and simple. These can be met by ensuring the aesthetic design and platform structure are consistent and clear. Co-design and development are crucial due to the difficulty in ensuring that online interventions are relevant to specific audiences in the constantly evolving digital landscape. The structures surrounding our joint adaptation of an existing intervention meant that not all the changes suggested could be implemented. Future work should explore the impact of different participation frameworks when coproducing interventions with young people.

## Introduction

There are estimated to be more than 2 million mental health apps worldwide, with a market value of more than US $8 billion in 2025 [1,2]. While the popularity and growth of these are due in part to ongoing digital wellness trends [3], there is also an increase in need. Worldwide, there is a mental health care crisis with services unable to cater to those who require access [4]. This is especially the case for young people, who have had an exponential increase in mental health challenges in recent years and have been particularly affected by the COVID-19 pandemic [5]. As evidence of this, 7.5% of UK university students declared a mental health diagnosis in 2023, compared to 0.7% in 2011 [6]. Recent research indicates 57% of university students have a mental health condition [7].

In addition to the increase in need for digital mental health interventions (DMHIs), there is also a strong argument for their use for young people. Young people aged 18‐25 years have grown up in a digital landscape. Overall, 79% of youth globally are online, compared to 65% of adults [8]. Additionally, young people have increased barriers to using mental health services due to a lack of mental health literacy, that is, understanding and knowledge of mental health conditions, how to get help, and how to prevent worsening of symptoms [9]. Hence, the accessibility of mental health support that can be accessed through a mobile phone or laptop could improve the well-being of young people [10].

However, there is a huge variety of apps that are designed to improve young people’s mental health, with inconclusive research evidence on their effectiveness [1]. Only apps that are based on cognitive behavioral therapy, supplemented with therapist contact, have been shown to be effective [11]. There are many contextual factors as to whether people engage with DMHIs, including the relevance of the content, the length of the activities, and ease of integration into daily life [12]. Use of digital mental health apps is rarely sustained over time [13].

A solution to this is to include young people in the development and evaluation of DMHIs [1]. This has been argued to be particularly important in increasing the use of mental health support for marginalized and underserved groups, enabling product developers to identify aspects of interventions that cause users to engage or disengage [9]. There has been a recent growth in studies reporting on the codevelopment of interventions in a research setting. However, this has occurred alongside increasing concerns about the quality of the co-design process, with arguments that the amount that young people can truly impact an end product is always limited by time and budget constraints [14,15].

This paper describes the advice given by young people, namely university students, in the process of adapting a preexisting web-based digital tool (i-Spero) for use in the university setting in the United Kingdom. This was conducted as part of the UK Research and Innovation–funded Nurture-U project that explored the whole-university approach to student well-being [15,16]. The i-Spero is a mental health symptom monitoring and care planning tool which, prior to its use in Nurture-U, had been implemented in UK National Health Service (NHS) settings and shown to be effective in supporting students in a Canadian university setting [17]. The Nurture-U research team worked collaboratively with the project’s student advisory group (SAG) to maximize the appeal, relevance, and usability for university students in the United Kingdom as part of a 3-year feasibility project. This study aims to present an inductive analysis of meeting notes and recordings of the joint adaptation process, aiming to highlight what aspects of DMHIs our advisors valued, alongside details on what changes were and were not implemented as a result. We will then reflect on this process in the discussion, highlighting the challenges in codeveloping DMHIs with young people. The hope is that this will inform future mental health interventions in this age group.

## Methods

### Existing Product for Development

**Figure 1.**
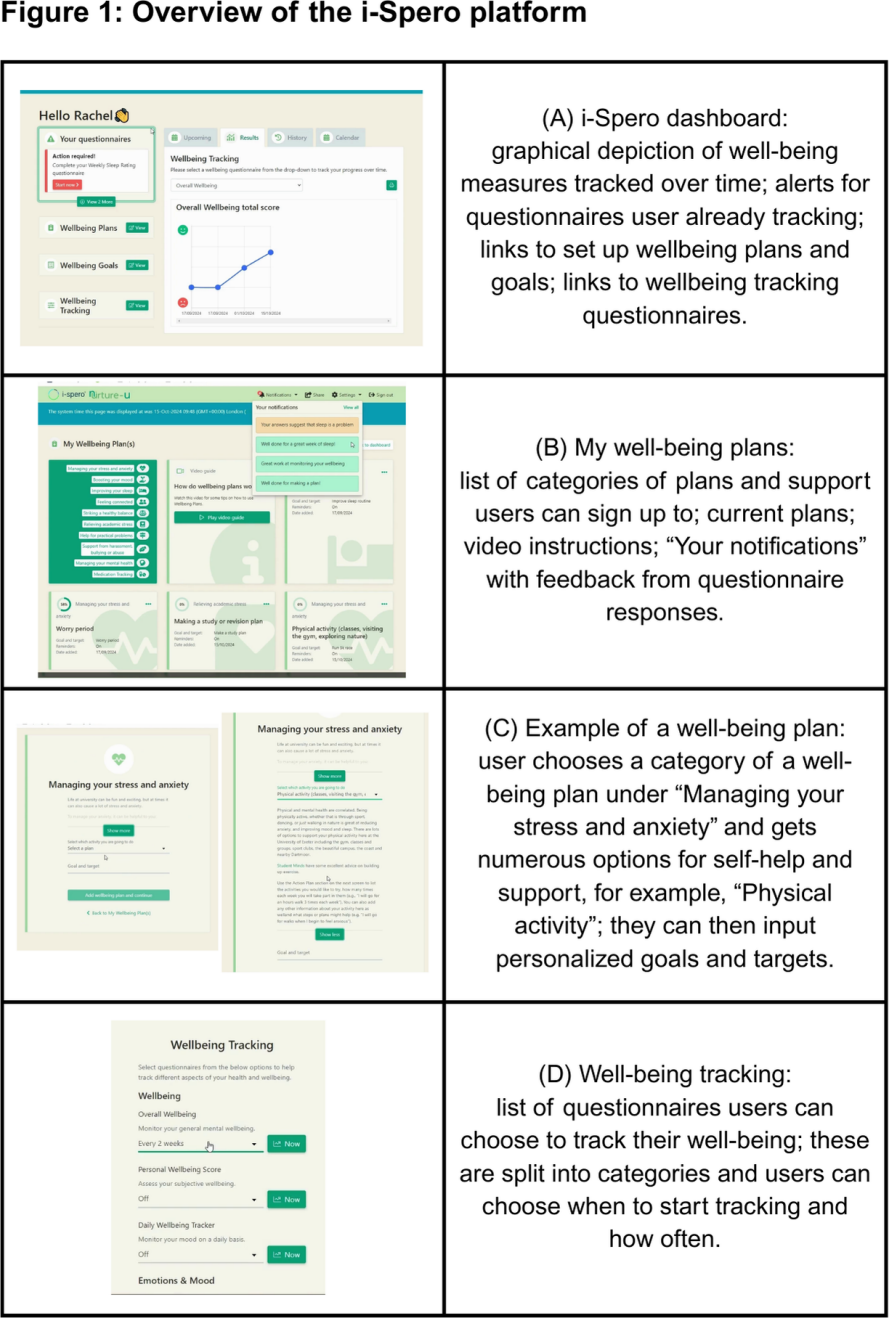
Overview of the i-Spero platform.

i-Spero a web-based platform developed by P1Vital as a digital tool for mental health, contains mental health and well-being measures and allows users to complete these and monitor symptoms over time [16]. It was developed to help individuals work with clinicians, for example, general practitioners (GPs), to identify the effect of antidepressants and predict response levels as early as possible. Figure 1 shows the key areas of the platform, in the format used in the Nurture-U project. Figure 1A shows the user dashboard with graphs tracking well-being over time, using responses to questionnaires chosen by the user. The platform links users’ answers on well-being questionnaires with in-built “well-being plans” (Figure 1B), which are categorized according to different types of support. Users can either choose their own well-being plans or i-Spero will suggest well-being plans based on their answers to the questionnaires (see “Notifications” in Figure 1B). The well-being plans also allow users to create “goals” and “actions” to improve their well-being (Figure 1C). Users can choose what to track and how often (Figure 1D). For example, if a user’s answer on a mood questionnaire indicates low mood, the platform will show a message recommending well-being plans to alleviate mood, such as making social connections, exercising, or mindfulness exercises. Additionally, users can share their information through dashboards with health care professionals or friends and families.

### Initial Joint Adaptation Process With the Nurture-U SAG

The Nurture-U SAG played a crucial role from the start of the project and fed into all aspects, from branding, content development, data collection, marketing, analysis, and dissemination. Students were recruited through university newsletters and groups from the 6 partner universities in the United Kingdom. There was no selection process for joining the SAG. Instead, on consenting to participation, students joined a mailing list where opportunities to input into different parts of the project were advertised. If the student advisor had capacity and an interest in that activity, they would email the group lead, who would add them to a working group for that project. Student advisors were paid £16 (US $21.48) an hour for attendance at meetings and work done outside of meetings. More details on the set-up and impact of the Nurture-U advisory group have been described elsewhere [18].

The opportunity to support the adaptation of i-Spero to Nurture-U was advertised to the SAG in December 2021 with an initial introduction to the tool from P1Vital. Four 2-hour meetings were held weekly in February 2022 on the following areas: in-built questionnaire design, well-being plans, notifications and messaging, flow, and evaluation. An average of 26 (SD 2.6) students attended per group. Following these intensive meetings, the research team adapted the design specifications using student feedback, and P1Vital implemented the Nurture-U adaptations to the i-Spero package for initial user testing.

Using Shier’s participation model [19], as implemented in the scoping review by Larsson et al [15] in this area, this was a Level 4 participation framework. Shier’s model has 5 levels, ranging from 1=listening to 5=sharing power. In our study, student advisors provided pointers and suggestions for edits to i-Spero, an existing product, and these were taken away by the research and software development team, who made the final decision on implementation according to practicality and relevance.

### Development of University-Specific Content With the SAG

The development of i-Spero for the university setting was motivated by evidence that one of the key barriers to support for students is accessibility, with students reporting complex websites and disparate services [20]. To this end, a crucial part of the integration of i-Spero within universities was that it allowed personalized recommendations for university campus–specific support for students. This entailed 6 different iterations of i-Spero for the partner universities within Nurture-U. The project leads in each institution led on the compilation of this information and extensive testing to ensure links and contact details were up to date.

Additionally, the tool was developed with a research aim of collecting prospective mental health data from university students through the tool’s mental health tracking feature, as a companion to a large-scale student mental health survey [21]. Alongside the development for university settings in the United Kingdom as part of the Nurture-U project, there was development for college students in Canada through the U-Flourish project [17].

### User Testing and Feedback

The first prototype of i-Spero for Nurture-U was ready for testing by students and the research team in June 2022. This did not include all the questionnaires and well-being plans but allowed the students and researchers to experience the tool and provide feedback. Student advisors provided notes and comments in self-created Microsoft Word documents or Excel spreadsheets to the SAG facilitator (JD). JD synthesized these comments with the researcher team’s comments and sent them to P1Vital to complete their adaptations.

The development was complete in November 2022. Plans for branding and marketing were built with the SAG, and it was rebranded as “the Nurture-U Wellbeing Toolkit.” This was launched across the 6 Nurture-U partner universities in January 2023. The Toolkit was marketed through stalls on campus, newsletter bulletins, social media posts and advertisements, and emails to students.

As well as the broad marketing to all students, SAG members were specifically invited to test the Toolkit and provide qualitative feedback through an online focus group with Nurture-U researchers and the P1Vital team in July 2023.

This feedback led to the next iteration of the toolkit, which was available and promoted through the same avenues in January 2024. Again, SAG members were invited to test the toolkit and provide qualitative feedback in April 2024. P1Vital implemented the suggested changes, and the final iteration to be tested in the Nurture-U study was launched in September 2024. As stated previously, evaluation and analysis of the user data to establish the acceptability of the software is currently ongoing, with the latest information from this process available on the Nurture-U website [22].

### Qualitative Analysis

The contents of 12 documents were analyzed. In total, 4 of the documents contained meeting notes from the initial development stage, with a range of 6‐9 pages of text, and the remaining documents ranged from 1‐9 pages of user feedback. A general inductive approach was implemented [23]. This is a method that aims to condense raw data into a concise summary for evaluation purposes. It is purely data-driven, with a bottom-up approach creating categories from participant quotes, using these to derive themes relating to the research question. This inductive process aimed to allow an overarching description of the student feedback across the different data sources.

Initial codes and categories stuck closely to the wording from the documents, for example, “if given too many options then too hard to engage” or “don’t want it to feel like extra work.” The initial inductive coding was completed independently using NVivo (version 14; Lumivero) by researcher JD and Nurture-U student advisor NA. JD and NA then compared initial codes, and then these were synthesized by JD into broader themes and checked by NA. This method of independent parallel coding is commonly reported in qualitative analysis as a method of ensuring rigor and trustworthiness [24]. As the author, JD had a research team perspective and NA had a student advisor perspective. This allowed for reflexive discussions about positionality and an exploration of the impact that had on the coding.

### Ethical Considerations

Ethical approval for the collection and publication of data related to the SAG was granted by the University of Exeter Centre for Life and Environmental Sciences Ethics Board (application ID 493946). All participants provided informed consent prior to taking part in the study. They were given clear information about the purpose of the research, what participation involved, and their right to withdraw at any time without penalty. All data were collected and processed in accordance with the General Data Protection Regulation and University of Exeter data protection policies and were only accessible to the research team. Personal identifiers were removed at the point of collection, and anonymized documents were used for analysis. Results are reported in aggregate form to ensure that individual participants cannot be identified. Participants were informed of their rights under the General Data Protection Regulation, including the right to access, rectify, or request deletion of their data.

## Results

### Overview

The Nurture-U student advisors provided input and feedback on the development of i-Spero in 4 different stages of its development over 2 years. A summary of changes that were and were not able to be implemented can be found in Textbox 1.

Textbox 1. **Implemented and nonimplemented student feedback.**

**Student-led changes implemented into the final product**

- All language suggestions for messages and notifications.
- Addition of student-designed questionnaires relevant to student issues.
- Changes to the color and layout of the interface.
- Addition and restructuring of well-being plans to enhance user experience.
- Addition of emojis and motivating messages.
- Inclusion and emphasis on methods of self-help.

**Student suggestions not implemented by the research or software team**

- Language suggestions for standardized research questionnaires.
- Removal or shortening of standardized research questionnaires.
- Addition of an area within the platform where users can connect with other users.
- Restructuring of software so that support can be accessed without well-being plans.
- Addition of motivational tools, such as a growing tree, or linking with other apps.
- Removal of signposting to medical and university settings.

**Figure 2.**
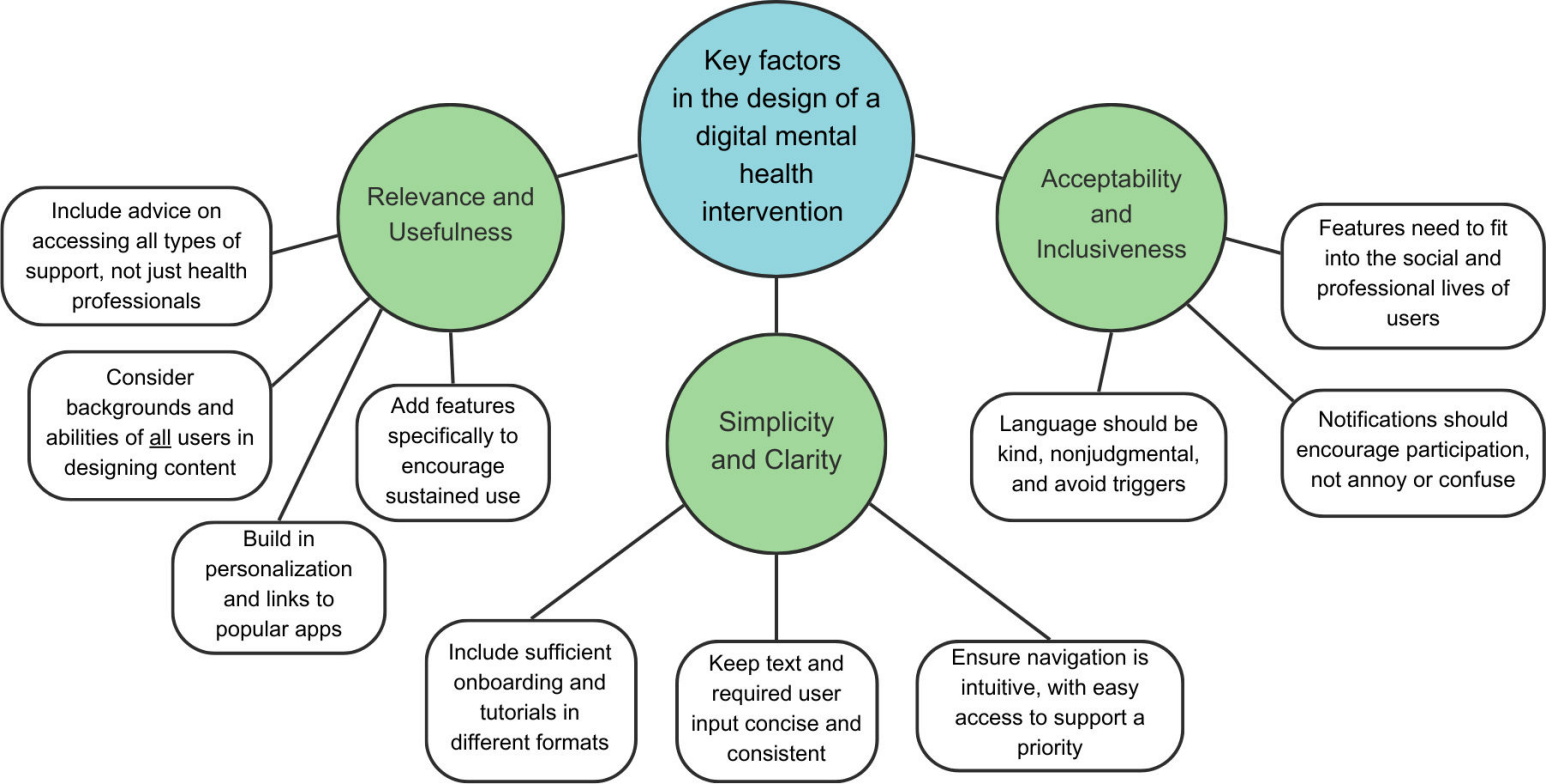
Core themes in i-Spero codevelopment.

Analysis of development notes and documents identified 4 key areas where students consistently highlighted the need for improvement: “Relevance and Usefulness,” “Simplicity and Clarity,” and “Accessibility and Inclusiveness.” These are summarized in Figure 2.

### Relevance and Usefulness

This theme encapsulates feedback from the student advisors that highlighted that the platform essentially had to be something that students actually want to use. The feedback was mostly positive; advisors highlighted the ease of navigation and appealing design, noting that the platform is accessible across different devices. Student advisors gave specific feedback from the perspective of young people on what aspects of the content were useful and what was not. For example, while they understood that repeated signposting to medical or university services in different areas of the platform may be necessary to ensure people get the care they need, they argued that this was not useful, as students already know this is where they can go for help.

It would be good to have specific apps and resources and not just to signpost people to Wellbeing Services and GP.

They argued that students will be attracted to the platform because they are struggling to find where to get help or have a reason to want to avoid traditional support options. Hence, student advisors argued that:

The hierarchy should change for suggestions for contact. So start with (1) friends and family, (2) wellbeing services, and then (3) GP.

Additionally, advisors fed back that vague language or advice was not helpful and could be found anywhere. They repeatedly highlighted where unnecessary words could be deleted. They also helped tailor advice so it was relevant to student contexts, for example, academic study. They also advocated for considering students from all backgrounds, for example, making alcohol-related content optional and creating specific content for underrepresented students, for example, those who are neurodiverse, or for parents or carers. Students advising on the initial iteration argued, for example, for the inclusion of a personality questionnaire, as something that would appeal to students at an age when they are learning who they are in the world.

The structure of the platform, tracking and setting goals, had mixed responses according to their relevance in the student context. Some advisors reflected that its usefulness lay in providing a bigger picture of emotions and mental health symptoms over time. However, the weekly *“*repetitive*”* questionnaires, some advisors argued, *“*felt like research*”* rather than something of use to them. They described the ability to set goals and tick them off as encouraging but reported little in the platform that encouraged sustained use and *“*ongoing management*”* of these goals when they were in place.

I don't find much reason to look back at the wellbeing plans I set myself.

Some advisors suggested direct feedback, which congratulated users on sustained use.

Could there be some sort of visual feedback so that people have a sense of accomplishment when they complete a questionnaire? Because at the moment there is no positive feedback until you have filled it out over time. For example, there could be a tree or sapling that grows into a forest?

An often-repeated aspect of feedback was to provide more areas for personalization, so users could individualize the content according to their preferences and experiences. Many advisors advocated for space in the platform for journaling and writing notes, creating a record of why they felt a certain way at different points over the academic year. Additionally, the ability to set their own reminders, notifications, and well-being plans so that they were experiencing the functioning in their own language and contexts was requested. They also repeatedly asked for the ability to link to apps they already use (eg, Spotify or exercise apps), or to include a social aspect such as a forum or the option to connect with other users and motivate each other to use the platform.

 Could this Tool connect people together to talk about their mental health?

Another factor that student advisors identified as important in young people finding the platform useful was that it should be motivating and positive. The danger that student advisors highlighted was that in tracking mental health over time and signposting to support, there was a risk that the platform would be repeatedly telling people they feel depressed or anxious, and that could be “disheartening.” The largest impact that the student advisors made in adapting the platform was in rewriting all the notifications, messaging, and well-being plans to provide encouragement to users regardless of their outcomes on the questionnaires. Advisors described a nuance to this where positive messaging could be “slightly patronizing” and hence more practical messaging and advice would be most relevant to their context.

Motivational messaging might be condescending. We could have testimonies or real-life stories of people who use the Tool – this is more likely to make a user feel better rather than something overly positive.

They emphasized how students were mostly young people who appreciated being “treated like an adult” as they began to build their lives away from home, and that the platform content needed to reflect this.

### Simplicity and Clarity

The second ongoing aspect of student advisor feedback on the intervention was to increase its simplicity of use and clarity of the information provided. Student advisors stated that anything that felt like “extra work” would not appeal to students, who already have enough to juggle with academic work and navigating new social situations. Initial feedback highlighted a lack of clarity in how to use the tool.

It’s very fiddly and complicated to use. The wellness plans still confuse me, and it doesn't seem easy to identify or access resources.

While guidance was provided in later iterations in the form of video onboarding on sign-in and links to external, more detailed video tutorials, this feedback continued. Advisors argued that navigation around the platform should be intuitive. They highlighted the need for improvements in how the information was organized, such as increasing the directions on the home page, moving from having “history” and “upcoming” areas to view activity on the platform to having an ongoing timeline, and being able to mark all notifications as read in one click. These were all implemented by the software team.

Consistency was a key area that students highlighted as important to usability, for example, scales on graphs all going the same direction. Another example given was that postquestionnaire messaging could be contradictory, so if you answer indicating, for example, low mood on one questionnaire but good sleep on another, you may receive contradictory positive and negative messages about your well-being.

Is there a way they can “trump”’ the other messages so they don’t contradict each other?

This solution, also implemented by the software team, was to have a hierarchy of messaging, where messages in response to mental health questionnaires (ie, mood or anxiety) would take precedence over lifestyle factors (eg, sleep or social connections).

In relation to the tracking element of the platform, an emphasis was made on avoiding burden for student users.

Won’t long questionnaires mean the students will get bored?

This led to the development of tracking questionnaires designed by student advisors, which involved branching elements where further questions were only asked in response to certain responses to initial brief questions.

This could start with a general question (e.g. “I’ve been feeling stressed a lot lately” rated from strongly disagree to strongly agree). You could then have follow up questions depending on your answer to the first general question, so if you indicate you are stressed you could have a list of things that might be causing you stress (for e.g. “what causes you to feel stressed?” with various categories (finance, relationships, academic work, cu), about frequency “how many days this week have you felt stressed?”, or “what do you to deal with stress?”. This could help find out the cause of stress and come up with coping strategies.

Additionally, students designed the user satisfaction questionnaire so that it was as concise as possible. However, the fact that the platform was embedded in a research project meant that certain standardized mental health questionnaires needed to be retained, for example, the Patient Health Questionnaire-9 (PHQ-9) and Generalized Anxiety Disorder 7 (GAD-7), and the wording of these questions could not be changed. The inclusion of these, alongside the additional questions designed by student advisors, led to a total of 33 questionnaires that student users could engage with. The student advisors who led the initial development of the questionnaires argued that all should be clearly labeled as to what they measure, including citations and links to sources, to enable students to engage with the ones that work best for them. Those that were designed by student advisors were named “student co-created.” However, student advisors who tested the later iterations of the platform expressed some confusion about the number of questionnaires and how they were labeled.

The well-being plan function, where users could choose certain areas to work on (eg, managing their mood and academic stress) and then access specific information and set goals, was seen as too complicated for many:

I feel like the well-being plans are just extra work, so I did not want to do it.

Advisors argued for a separate page where resources and signposting could be read outside of the well-being plan format for ease of access. This was implemented by the software team. The type and level of support wanted from the intervention was a subject of debate, with some advisors wanting “comprehensive” and detailed psychoeducation, as that is what they saw as the function of the platform, while others wanted less information and text and instead practical and accurate signposting for different areas of need. Advisors argued that text should be broken into paragraphs and boxes to reduce “overwhelm.” This was implemented by the research team.

### Acceptability and Inclusiveness

Finally, student advisors were central to ensuring the design was acceptable to the student population, the majority of whom were under 25 years of age. A key factor in this was the aesthetic design and “feel,” with initial feedback on the version that had been used in the NHS declaring it “outdated,” “medical” and “bland.” The Nurture-U brand colors [22] were decided upon as a potentially recognized design that was distinct from the participating universities and the NHS. Given the many iterations of the platform, with different versions for different campuses entailing many different stakeholders providing input, there were initial areas of inconsistency or errors in the language used, which student advisors highlighted as off-putting in an intervention targeting well-being. Hence, while student advisor input was built in for the purpose of ensuring relevance to students, they also provided basic error checks, which were extremely valuable.

A big part of the feedback on the acceptability of the tool for young people was the role of the notifications for engaging in the questionnaires and the well-being plans. Advisors provided the wider context of the use of the platform: students live busy social lives where their phones and laptops are often on display, and hence the subject line of reminder emails and texts needed to be neutral or *“*vague*”* to protect people’s privacy. Notifications needed to be unintrusive but also engaging to encourage students to prioritize the platform over their other tasks.

We could make [the reminder message] more positive: “We’ve missed you! Click here to do your wellbeing questionnaire now”

Notifications containing a list of *“*long, formal questionnaire names*”* were anxiety-provoking and would be identified as another burden in a busy period of academic work. These contextual descriptions of the target user allowed for changes, which it was hoped would encourage higher levels of engagement from students.

However, student advisors also highlighted that it was not only young people as a broad audience who would be using the platform, but young people who were likely to be experiencing challenges with their mental health.

Make it more organised and aesthetic - If a person is struggling, they don’t want red bold text or long paragraphs. It should feel like a safe space.

There were concerns from some of the advisors with lived experience of depression and anxiety that when they were experiencing more severe symptoms, they would not have had the energy and motivation to engage with tracking and goal setting. While some aspects to deal with this challenge also arose in the previous theme surrounding ease of access to resources, advisors also specifically discussed the “tone” of the information given. They asked for the removal of moral descriptions of behaviors as good or bad, advising that language should be neutral and warm:

We don’t want the questions to be judgemental or triggering. For example, people may have different perceptions of what constitutes “good” sleep or diet depending on their lifestyles, experiences of eating disorders, cultural backgrounds. This is why more generic questions that people can tailor to their own personal experiences might work best. The language should be kind and inviting.

Language should also be neutral and warm in describing lifestyle factors such as sleep or diet, which are individual and may vary according to background and experiences. They also highlighted instances of tonal mismatch, where messages starting with “hello” were too informal for serious notifications about mental health.

Student advisors argued that users would be logging on to the platform specifically for support, and, hence, ease of access to this support is of high importance. As discussed in the theme relating to simplicity, students argued that the well-being plan interface was a barrier and that students should not have to “commit to a plan” to get support. Similarly, in order to engage those who are having difficulties with their well-being and mental health, advice should be “reassuring”: not telling students what to do but “talking to them on a level.” Student advisors highlighted specific language changes that could achieve this, for example, normalizing experiences and pitching advice as “beneficial to lots of people,” and not promising an unachievable cure but highlighting “useful steps” toward an improvement in symptoms. Finally, student advisors highlighted several reasons why users may have difficulty understanding certain terminology in the advice sections, whether it was a lack of mental health literacy, cognitive challenges due to poor mental health, or not having grown up in the United Kingdom. Hence, there were many aspects of the content where they suggested simplifications or the need for additional definitions to enhance accessibility. These were all implemented by the research team.

## Discussion

### Principal Findings

In the joint adaptation of the i-Spero platform for university student users, 3 key concepts were identified as crucial to designing DMHIs for young people. These core ideas of relevance and usefulness, simplicity and clarity, and acceptability and inclusiveness should be central to all elements of interventions, from the interface to the content to the notifications. While we were unable to implement all suggested changes within the Nurture-U project, this study aimed to specify what young people want from digital mental health support to inform future design.

Where the Nurture-U student advisors made the most impact in adapting i-Spero was in highlighting where information and support needed to be added for underrepresented groups and where edits needed to be made to remove burden from student users. These 2 areas of feedback, however, became contradictory over the development process: including questionnaires and well-being plans that covered the needs of all students necessitated more text and information, which in turn created more content that student advisors identified as overwhelming. This is the key challenge in developing online mental health support: trying to appeal to all when everyone’s needs are different [25-28]. There is a difficult balance, identified both by our advisors and in the wider literature, in making information comprehensive but also digestible [27]. What the advisors highlighted as key to engagement is not so much the amount of information contained in an intervention, but how it is presented and how easy it is for users to find what they need. A recent interview study with teenagers found that while a clinical approach to presenting information, such as that on NHS websites, promotes trustworthiness, it can be intimidating and difficult to read [27].

Student advisors overhauled the language used in the intervention. We were not surprised that they would highlight the need for clarity; clear and nonjudgmental language is well-known to reduce mental health stigma and promote support [29]. What perhaps was novel to the research team was the advisors’ requirements for motivational language, positivity, and interaction to increase engagement. In a competitive online landscape, it is the “gamification” of apps and content, defined by creative thinking and activation, which encourages use [30]. To adapt to these suggested changes in mood and aesthetic of the intervention, we implemented all edits to language and messaging, including brighter colors, and minimized the use of buttons and extensive scrolling to achieve tasks. However, there were features in the original platform design, such as the format of the well-being plans and the research design, such as the language in the standardized questionnaires, which we were not able to adapt as the student advisors may have wished. We will see the impact of keeping these functions as we analyze the user data [22].

Another aspect of engagement that we had not anticipated was how much student advisors asked for personalization of the intervention. Research has shown that personalization of the mode of delivery of online health information can increase website satisfaction and information recall of participants, and that this effect was particularly strong with younger people [31]. However, research into how to personalize digital health interventions is relatively recent; where personalization is built in, it is usually for content over format, and there is not yet evidence on the effect of different levels of personalization on outcomes [29]. As personalization through algorithms is becoming more ubiquitous on social media, in search engines, and music and video streaming sites, this is something that is likely to become increasingly necessary to ensure engagement in DMHIs.

Another aspect that the research team was unable to change, despite the student advisor’s recommendation, was to step back from signposting users to professional services for mental health. Highlighting the need to access GP or university well-being services was crucial for managing the associated risks of mental health difficulties, especially as the target user would be young people who are likely to be away from home for the first time. Research shows that many young people are not aware of where to get professional help for mental health difficulties [32]. However, advisors argued that student users would not only be aware of these options but actually may be accessing the platform specifically looking for other ideas to support their mental health. This mirrors Biddle et al [33] review of young people accessing support for suicidal thoughts online, where being referred back to a doctor was not only frustrating but also damaging to those in crisis: seeing this as the only option when it has not worked previously makes people think they cannot be helped. Those who develop mental health interventions must not see the user as accessing the intervention in isolation and be aware of the real-world contextual influences and experiences on the target user [27]. There is an ethical discussion as to whether apps that are targeted as mental health support should have a duty of care for users [34]. However, the context of this study, abiding by research ethics and university principles, meant that we needed to ensure all student users were aware of professional services and how to contact them if needed.

In conducting this study and reflecting on the findings in the context of previous literature, there is evidence of marked similarity in what young people want from digital mental health support between multiple different studies and reviews [12,14,15,33]. What this suggests is twofold: first, researchers working in this space need to do more to learn and build on preexisting research when they are designing and conducting projects in this space [35]. However, and conversely, this reflects the fact that the internet has created a rapidly changing social and political landscape where the factors that make a platform accessible and sustainable are constantly evolving [36]. One reason researchers repeat these elements of codevelopment is that while the overarching advice from young people may be the same, for example, ease of use or age-appropriate language, the specifics of this may vary according to context, be it geographical or cultural [37]. Including the target audience, especially in interventions aimed at young people, in mapping out the intervention theory and development of prototypes is crucial to creating DMHIs that have sustained benefits [28,35].

We placed our joint adaptation of the i-Spero intervention as a level 4 within Shier’s participation framework: the student advisors were involved in the decision-making, but they did not share the responsibility for the ultimate decision (level 5) [15]. Shier emphasizes that his framework is not hierarchical, that different levels of participation are appropriate in different contexts [15]. However, there are increasing concerns that the growing prevalence of coproduction occurring without a critical and evaluative lens on power and decision making may be detrimental to the original aim of producing “socially robust” research outcomes [19]. In the case of DMHIs, this specifically means that placing limits on the extent of coproduction results in a product that may not fit user requirements. What our study highlights further, however, is the often-competing agendas that are at play in codeveloping DMHIs, especially in the context of research. First, where software developers have existing designs or researchers have existing research aims, this creates areas where those with lived experience have to be told “no.” When considering working relationships, this builds a level of power imbalance where the advisors may lose faith in the project and their motivation to contribute wanes. A fully coproduced project, where people with lived experience colead or even lead the process of designing and delivering a DMHI, would avoid these challenges, but would take far more time and resources. Further research in this field would be to compare the impact of DMHIs that are fully coproduced with people with lived experience, as compared to ones which only have user-testing elements, both on mental health outcomes for users and costs and resources.

There were strengths in our extended joint adaptation process of i-Spero with university students, including the rigor and transparency in the researcher and advisor feedback process [18]. Additionally, a reflection of the process as we have presented in this paper is crucial for effective participatory research [38]. However, there were some limitations. First, while the student advisors were the target audience in that they were university students, they had joined the advisory group because they had an interest in student well-being, not necessarily because they themselves were experiencing mental health challenges or seeking support. Hence, they may not have been the students that the intervention was designed for. Another limitation was that we only used focus groups or written feedback; more inventive or creative approaches could have garnered more detailed insights [15,28]. Additionally, as discussed above, while i-Spero was initially developed with co-design and research methodologies, by the time we were adapting the platform, there were some aspects that could not be changed within the research timeframe and budget.

### Conclusions

To conclude, inductive analysis of our records of the joint adaptation process generated 3 key themes for designing DMHIs for young people: “Relevance and Usefulness,” “Simplicity and Clarity,” and “Accessibility and Inclusiveness.” While these are concepts that have been identified in other studies, it is important to recognize that they are also constantly changing entities. Hence, co-design with users from the inception of a digital intervention idea is key to ensuring effective and sustainable digital mental health support. However, there is a need for more research exploring the impact of different levels of user participation in codevelopment on intervention outcomes.

## Abbreviations

DMHI: digital mental health intervention
GAD-7: Generalized Anxiety Disorder 7
GP: general practitioner
NHS: National Health Service
PHQ-9: Patient Health Questionnaire-9
SAG: student advisory group
